# Continuous subcutaneous levodopa and foslevodopa infusion therapy for motor symptoms of Parkinson disease: A systematic review of observational studies

**DOI:** 10.1097/MD.0000000000049597

**Published:** 2026-07-03

**Authors:** Aasim Ali, Waqar Ahmed Cheema, Muhammad Shamoon, Hammad Azam, Dansih Yousuf, Anousha Tanveer, Wajeeha Asif, Fiza Nisar, Labeeba Abdul Ghafoor, Muhammad Talha, Muhammad Abdullah Ali, Usama Saleem, Muhammad Asad Shabbir, Armaghana Abdullah, Mukesh Sharma

**Affiliations:** a Neurology Department, Allied Hospital Faisalabad, Faisalabad, Pakistan; b Internal Medicine Department, Allied Hospital Faisalabad, Faisalabad, Pakistan; c Neurology Department, Dhankuta Multiple Campus – Tribhuvan University, Dhankuta, Nepal.

**Keywords:** ABBV-951, continuous subcutaneous infusion, foslevodopa/foscarbidopa, levodopa, motor fluctuations, ND0612, observational studies, Parkinson disease, real-world evidence

## Abstract

**Background:**

Parkinson disease affects approximately 6.1 million people worldwide. Although levodopa remains the most effective symptomatic therapy, long-term oral treatment commonly leads to motor fluctuations and dyskinesias that impair quality of life. Continuous subcutaneous levodopa infusion therapies have emerged as less-invasive strategies to provide continuous dopaminergic stimulation and reduce motor complications.

**Methods:**

This systematic review evaluated the efficacy and safety of continuous subcutaneous levodopa infusion therapies, including ND0612 and foslevodopa/foscarbidopa (ABBV-951), in advanced Parkinson disease (APD). Following Preferred Reporting Items for Systematic Reviews and Meta-Analyses 2020 guidelines, PubMed, Scopus, Web of Science, and Google Scholar were searched from inception to April 2026. Observational studies assessing continuous subcutaneous levodopa infusion in adults with APD and motor fluctuations were included. Ten studies comprising 867 patients met the inclusion criteria.

**Results:**

Across studies with follow-up up to 36 months, continuous subcutaneous infusion reduced daily OFF time by approximately 2.0 to 3.5 hours and increased ON time without troublesome dyskinesia. Quality of life scores improved by 5.6 to 10.8 points on Parkinson Disease Questionnaire measures. Infusion-site reactions (ISRs) were the most common adverse events, occurring in 42% to 92% of patients, though most were mild to moderate. Severe ISRs leading to discontinuation occurred in 5% to 12% of patients. ABBV-951 demonstrated numerically lower ISR rates than ND0612 in real-world settings. Systemic adverse events were generally comparable to oral levodopa therapy.

**Conclusion:**

Observational evidence supports continuous subcutaneous levodopa infusion therapies as effective options for reducing motor fluctuations in APD, with sustained benefits reported up to 36 months. ISRs remain the principal tolerability concern, but severe discontinuation-related events are uncommon. Further comparative and long-term studies are needed to better define safety, efficacy, and real-world applicability across diverse populations.

## 1. Introduction

Parkinson disease (PD) is the second most common neurodegenerative disorder worldwide, with a global prevalence of approximately 6.1 million individuals in 2016, a figure projected to exceed 12 million by 2040.^[1]^ The disease affects more than 1% of individuals aged 65 and above, with incidence rising sharply with advancing age.^[2]^ PD is characterized by progressive loss of dopaminergic neurons in the substantia nigra pars compacta, resulting in the classic motor triad of bradykinesia, rigidity, and resting tremor, along with a broad spectrum of non-motor symptoms.^[3]^

Since its introduction in the 1960s, levodopa has remained the most effective symptomatic therapy for PD, producing robust improvements in motor function that translate into enhanced quality of life and reduced disability.^[4]^ However, the chronic use of oral levodopa is complicated by the predictable development of motor fluctuations and levodopa-induced dyskinesias. Within 4 to 6 years of treatment initiation, approximately 40% to 50% of patients experience wearing-off phenomena, and up to 80% develop motor complications after 10 years of therapy.^[5,6]^

The concept of continuous dopaminergic stimulation emerged from recognition that pulsatile levodopa delivery contributes to the development and persistence of motor complications.^[7]^ This pathophysiological rationale led to the development of device-aided therapies capable of delivering dopaminergic medication continuously, including deep brain stimulation (DBS), continuous subcutaneous apomorphine infusion (CSAI), and continuous infusion of levodopa-based formulations.

While intestinal levodopa infusions (levodopa-carbidopa intestinal gel [LCIG]) have demonstrated efficacy, they require invasive surgical insertion of a percutaneous endoscopic gastrostomy with jejunal extension tube, which carries procedural risks and limits accessibility.^[8]^ In contrast, continuous subcutaneous infusion (CSCI) formulations offer a less invasive alternative that can be initiated and discontinued more readily. Two primary subcutaneous levodopa formulations have been developed and evaluated in clinical trials. ND0612 is a CSCI of liquid levodopa/carbidopa, delivered via a portable pump over 24 hours. Foslevodopa/foscarbidopa (ABBV-951, marketed as Vyalev®) is a prodrug formulation that provides continuous subcutaneous delivery of levodopa and became the first and only subcutaneous 24-hour infusion of levodopa-based therapy approved for the treatment of motor fluctuations in PD in October 2024.^[9]^

Recent systematic reviews and meta-analyses have synthesized the growing evidence base for these therapies, primarily focusing on randomized controlled trial (RCT) data.^[10,11]^ However, real-world observational studies provide essential complementary evidence on long-term effectiveness, patient selection, infusion-site reaction (ISR) management, and practical implementation in routine clinical practice. This systematic review synthesizes evidence from 10 observational studies evaluating continuous subcutaneous levodopa infusion therapies for motor symptoms in advanced Parkinson disease (APD).

## 2. Materials and methods

### 2.1. Study registration and reporting

This systematic review adheres to the Preferred Reporting Items for Systematic Reviews and Meta-Analyses 2020 guidelines.^[12]^ The review protocol was registered on the Open Science Framework (registration DOI: https://osf.io/mt368).

### 2.2. Eligibility criteria

Studies were selected according to the Population, Intervention, Comparator, Outcomes, and Study Design framework.

Inclusion criteria: observational study designs including prospective cohorts, retrospective cohorts, and open-label extensions; adult patients (≥18 years) with a clinical diagnosis of PD (UK Brain Bank or Movement Disorder Society [MDS] criteria) experiencing motor fluctuations despite optimized oral therapy; intervention with CSCI of ND0612 (levodopa/carbidopa) or foslevodopa/foscarbidopa (ABBV-951); reported outcomes including OFF time, ON time, quality of life measures, or adverse events (AEs); follow-up duration ≥4 weeks; and sample size ≥10 patients.

Exclusion criteria: RCTs – recent meta-analyses have synthesized RCT evidence^[10,11]^; case reports or case series with <10 participants; studies not published in English; duplicate publications of the same cohort (the most comprehensive or longest follow-up report was included); studies evaluating non-subcutaneous levodopa administration; and conference abstracts without sufficient data (except where noted).

### 2.3. Search strategy

A comprehensive electronic literature search was conducted in PubMed (MEDLINE), Scopus, Web of Science, and Google Scholar from inception through February 2026. The search strategy combined terms for PD (“Parkinson disease”[MeSH] OR “Parkinson disease”[tiab]), subcutaneous infusion (“subcutaneous”[tiab] OR “continuous subcutaneous infusion”[tiab] OR “CSCI”[tiab] OR “ND0612”[tiab] OR “ABBV-951”[tiab]), and observational study designs (“observational”[tiab] OR “cohort”[tiab] OR “real-world”[tiab] OR “open-label extension”[tiab]). Reference lists of included studies and relevant reviews were hand-searched.

### 2.4. Study selection and data extraction

Two reviewers independently screened titles, abstracts, and full texts against eligibility criteria. Disagreements were resolved through discussion or adjudication by a third reviewer. Data extraction was performed independently using a standardized, pre-piloted form collecting: study characteristics (author, year, country, design, sample size, follow-up duration), participant demographics (age, sex, disease duration, baseline OFF time), intervention details (formulation, dosing, infusion duration), outcome data (OFF time change, ON time change, quality of life scores, AEs, discontinuation rates), and funding sources.

### 2.5. Risk of bias assessment

The Newcastle-Ottawa Scale (NOS) was used for cohort studies, assessing 3 domains: selection of study groups (maximum 4 stars), comparability of groups (maximum 2 stars), and ascertainment of outcome (maximum 3 stars). For single-arm studies, an adapted NOS was used focusing on representativeness, ascertainment of exposure, and outcome assessment. Studies with scores ≥7 stars were considered high quality, scores of 4 to 6 stars moderate quality, and scores ≤3 stars low quality. Disagreements in risk of bias assessments were resolved through discussion.

### 2.6. Data synthesis

Given the anticipated clinical and methodological heterogeneity among the included observational studies – particularly in outcome measures, follow-up durations, and study populations – a narrative synthesis was planned as the primary analytical approach. A meta-analysis was deemed inappropriate due to this heterogeneity. This heterogeneity manifested in several domains: variations in outcome measures (OFF time in hours vs Movement Disorder Society-Unified Parkinson Disease Rating Scale [MDS-UPDRS] components vs global impressions), follow-up durations (1–36 months), study populations (de novo patients vs those switching from apomorphine vs mixed), and intervention protocols (24-hour vs 16-hour infusion, concomitant medication management), as pooling such heterogeneous data would yield effect estimates that are difficult to interpret and potentially misleading. Studies were categorized by intervention type (ND0612 vs ABBV-951) and by follow-up duration. Effect sizes were reported as presented in the original studies. Where appropriate, ranges of effect estimates were summarized across studies.

### 2.7. Certainty of evidence

The overall certainty of evidence was assessed using the Grading of Recommendations Assessment, Development and Evaluation framework for observational studies,^[13]^ with evidence starting as low certainty and potentially upgraded for large effect sizes or dose–response gradients.

## 3. Results

### 3.1. Study characteristics

The systematic literature search yielded 2891 records. After removal of duplicates and screening, 10 observational studies met inclusion criteria as shown in Preferred Reporting Items for Systematic Reviews and Meta-Analyses flow diagram. The included studies comprised 1 study evaluating ND0612 (n = 214)^[14]^ and 9 studies evaluating ABBV-951 (total n = 653 patients).^[15–22]^ Geographic distribution included North America (USA and Canada), Europe (UK, Germany, France, Italy, Spain, Portugal, and Sweden), and Israel. Follow-up durations ranged from 1 to 36 months.

### 3.2. Participant characteristics

Baseline characteristics of participants across included studies were broadly similar and representative of APD populations with motor fluctuations. The mean age ranged from 63.8 to 72.0 years, with a slight male predominance (approximately 60%–65%). Mean disease duration at treatment initiation was 9.8 to 15.2 years. Baseline OFF time averaged 5.2 to 6.2 hours per 16 to 18 waking hours when reported, indicating moderate-to-severe motor fluctuations despite optimized oral therapy.

The characteristics of included studies are summarized in Table 1.

**Table 1:** Baseline characteristics of included observational studies.

Study	Country	Design	Intervention	N	Mean age (yr)	Female (%)	Disease duration (yr)	Baseline OFF time (h/d)	Baseline UPDRS-III (ON)	Follow-up
Ellenbogen et al 2025^[14]^	Multinational	Prospective OLE	ND0612	214	63.8 (8.7)	38	10.2 (4.1)	6.2 (1.8)	NR	36 mo
Aldred et al 2023^[15]^	Multinational	Prospective, single-arm, open-label, phase 3	ABBV-951	244	63.9 (9.2)	40.2	10.7 (5.2)	5.9 (2.2)	23.5 (11.5)	52 wk
Jander et al 2025^[16]^	Germany	Retrospective cohort (day-clinic)	ABBV-951	24	72.0 (9.2)	50	13.7 (5.4)	NR	30 (28–43.5)*	9 wk
Nordera 2025†	Italy	Prospective case series	ABBV-951	20	70 (62–80)*	30	10.4 (5–17)*	NR	NR	3 mo
Hartig et al 2026^[17]^	Germany	Retrospective cohort	ABBV-951	58	71.4 (8.5)	39.7	13.3 (6.2)	NR	36.2 (15.5)	Real-world
Pinna et al 2025^[18]^	Italy	Retrospective, multicenter	ABBV-951	103	66.3 (9.2)	35	12.6 (5.3)	NR	NR	3 mo
Das et al 2026^[19]^	Canada	Ambispective longitudinal	ABBV-951	64	NR‡	NR‡	NR‡	NR	NR‡	1 mo
Desjardins et al 2025^[20]^	France	Retrospective cohort	ABBV-951	14	69.4 (6.6)	50	10.5 (3.7)	NR	54.5 (20.0)	3 mo
Cocco et al 2025^[21]^	Italy	Pragmatic, real-world	ABBV-951	21§	65.3 (10.8)	47.6	12.3 (7.6)	NR‖	39.1 (13.9)	20 wk
Jost et al 2026^[22]^	Multinational	Prospective, multicenter, real-world	ABBV-951	105	68.5 (9.5)	NR	12.1 (5.3)	5.2 (0.6)	NR	6 mo

NR = not reported, OLE = open-label extension, UPDRS = Unified Parkinson Disease Rating Scale.

* Values reported as median (range).

† Conference abstract.

‡ Demographic data not reported in abstract.

§ 23 enrolled, 21 completed.

‖ MDS-UPDRS Part IV item 4.3 (time in OFF): 2.38 ± 0.99.

### 3.3. Risk of bias assessment

Quality assessment using the NOS revealed that 8 of 10 studies (80%) were of high quality (score ≥ 7 stars), 2 studies (20%) were of moderate quality (score 4–6 stars), and no studies were rated as low quality. Common methodological strengths included representative patient cohorts, prospective design with complete follow-up, and objective outcome assessment using validated scales. Common limitations included lack of independent blinded outcome assessment, potential selection bias in retrospective designs, and single-arm design without comparator groups.

### 3.4. Reduction in OFF time

Reduction in daily OFF time was the most consistently reported efficacy outcome across included studies. The 3-year open-label extension of the BeyoND trial reported by Ellenbogen et al^[14]^ in 214 patients treated with ND0612 demonstrated a mean OFF time reduction of 2.81 hours at 36 months and a corresponding increase in good ON time of 2.79 hours. Notably, 67.5% of patients who entered the extension phase completed 3 years of treatment, supporting long-term durability.

For ABBV-951, the 52-week open-label phase 3 trial by Aldred et al^[15]^ in 244 patients demonstrated a mean OFF time reduction of 3.5 hours and a good ON time increase of 3.8 hours at week 52, with 56.1% of patients completing the study. The percentage of patients experiencing morning akinesia dropped from 77.7% at baseline to 27.8% at week 52.

In the German day-clinic study by Jander et al,^[16]^ MDS-UPDRS Part IV score (motor complications) improved significantly by 53% at week 9 (*P* = .0267), with the OFF time component (item 4.3) reduced by 75% (*P* = .0094). The levodopa equivalent daily dose (LEDD) increased by 55% (*P* < .0001), reflecting the transition from oral to subcutaneous therapy.

The real-world German cohort study by Hartig et al^[17]^ (n = 58) reported a significant improvement in ON-UPDRS Part III of 6.07 points (*P* = .0008). Clinician-rated effectiveness was 89%, while patient-rated effectiveness was 74%, revealing a meaningful discrepancy in perceived benefit. Notably, 33.3% of patients discontinued treatment within 4 weeks, with 63.2% of discontinuations due to patient preference rather than AEs or ineffectiveness.

The multicenter Italian study by Pinna et al^[18]^ comparing continuous subcutaneous foslevodopa/foscarbidopa infusion (CSFLI; n = 103) to LCIG (n = 129) demonstrated that both therapies were similarly effective in improving motor symptoms and quality of life. However, CSFLI required significantly more follow-up visits during the first 3 months (*P* < .0001) and showed a greater increase in LEDD compared to LCIG (*P* = .004). Patients initiated on CSFLI were younger, had shorter disease duration, and lower Hoehn & Yahr stage compared to LCIG patients.

The Canadian ambispective study by Das et al^[19]^ (n = 64) reported an OFF time reduction of 2.77 hours, ON time without dyskinesia increase of 2.07 hours, and ON time with troublesome dyskinesia reduction of 0.74 hours at 1 month. MDS-UPDRS Part III improved by 11.37 points, and Parkinson Disease Questionnaire (PDQ)-39 improved by 10.78 points.

The French study by Desjardins et al^[20]^ evaluated 14 patients switching from CSAI to foslevodopa/foscarbidopa. At 3 months, MDS-UPDRS Part IV (motor complications) significantly improved (*P* = .011), as did Part II (activities of daily living, *P* = .0498), PDQ-8 (*P* = .0244), Clinical Global Impression-Severity (*P* = .0197), and Neuropsychiatric Inventory (*P* = .0166). Notably, no patient discontinued treatment, suggesting excellent tolerability in this specific population.

The pragmatic Italian study by Cocco et al^[21]^ (n = 21 completers) reported a 40% reduction in OFF time (MDS-UPDRS item 4.3: 2.38→1.43, *P* < .05) and a 57% reduction in dyskinesia time (item 4.1: 1.42→0.61, *P* < .05) at 20 weeks. The foslevodopa dose increased by 37% (*P* < .05) and total levodopa equivalent dose increased by 46% (*P* < .05). The median time to reach final therapeutic dosage was 12 weeks, and patients with higher body mass index required a longer titration period (*P* = .025).

The multinational ROSSINI study by Jost et al^[22]^ (n = 105) reported statistically significant reductions at 6 months in OFF time (−2.8 hours, *P* ≤ .001), dyskinesia time (−1.8 hours, *P* ≤ .001), MDS-UPDRS Part III (−5.0 points, *P* = .002), Parkinson Disease Sleep Scale-2 (−5.2 points, *P* ≤ .001), and PDQ-39 (−5.6 points, *P* = .002).

### 3.5. Improvements in quality of life

**Figure 1.**
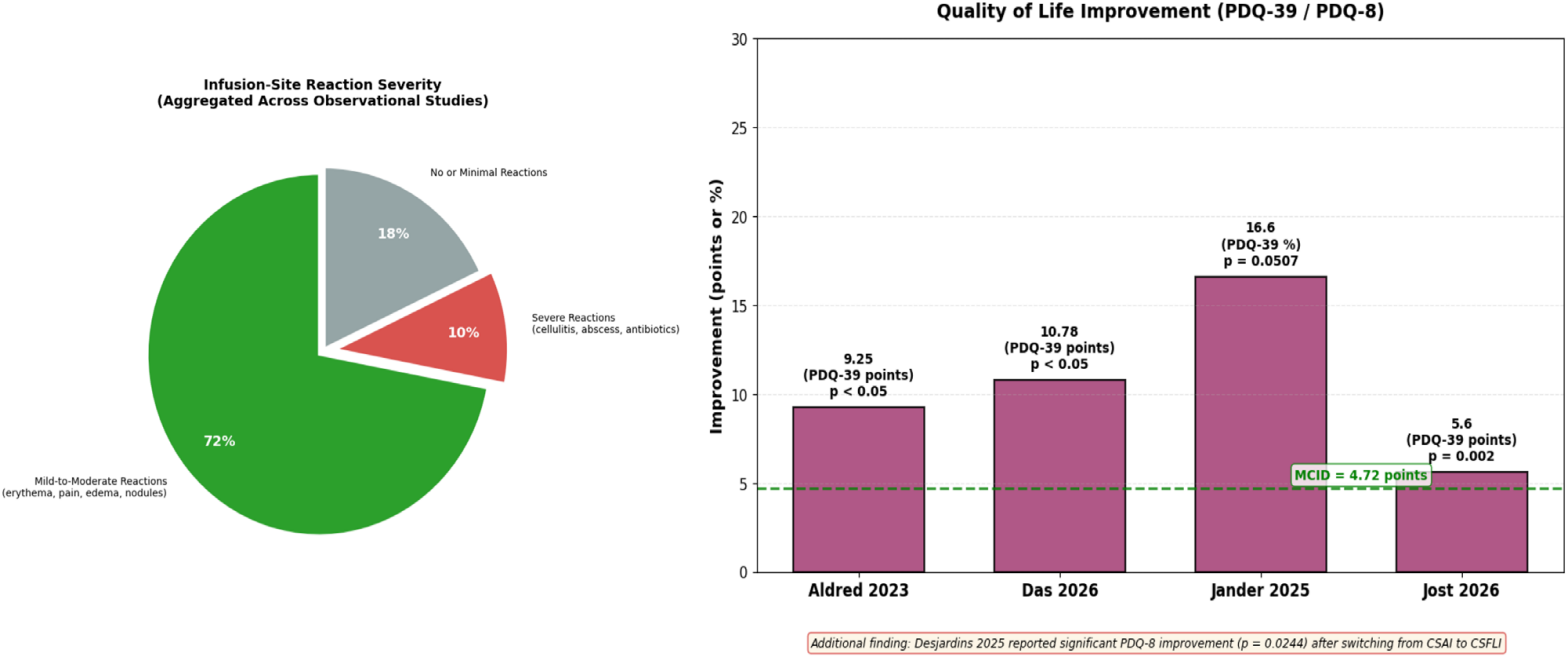
Safety and quality of life outcomes of continuous subcutaneous levodopa infusion therapies for advanced Parkinson disease (N = 867 patients). Quality of life improvement (PDQ-39/PDQ-8). All 4 studies exceeded the MCID threshold (4.72 points, green dashed line), indicating clinically meaningful improvement. *P* values are shown above each bar. Right panel: infusion-site reaction severity. Mild-to-moderate reactions (erythema, pain, edema, nodules) occurred in 72%, severe reactions (cellulitis, abscess requiring antibiotics) in 10%, and no/minimal reactions in 18%. Key insight: 24-hour needle change reduced skin reactions from 50% to 7.1% (Jander et al^[16]^). CSAI = continuous subcutaneous apomorphine infusion, CSFLI = continuous subcutaneous foslevodopa/foscarbidopa infusion, MCID = minimal clinically important difference, PDQ = Parkinson Disease Questionnaire.

Health-related quality of life, assessed primarily with the Parkinson Disease Questionnaire (PDQ-39 or PDQ-8), showed consistent improvements across studies. Aldred et al^[15]^ reported PDQ-39 improvements of 7.9 to 10.6 points at 52 weeks. Jander et al^[16]^ reported a 16.6% improvement in PDQ-39 total score (*P* = .0507), with significant improvements in mobility (*P* = .0015) and bodily discomfort (*P* = .0127). Das et al^[19]^ reported a 10.78-point improvement in PDQ-39. Desjardins et al^[20]^ reported significant PDQ-8 improvement (*P* = .0244). Jost et al^[22]^ reported a 5.6-point improvement in PDQ-39 (*P* = .002). These improvements consistently exceeded minimal clinically important difference thresholds where reported (Fig. 1).

### 3.6. Safety: ISRs

ISRs were the most common AEs across all observational studies. Critically distinguishing between mild-to-moderate and severe reactions is essential for clinical decision-making.

For ND0612 (Ellenbogen et al^[14]^), ISRs occurred in 92.1% of patients in year 1. However, severe reactions leading to discontinuation occurred in only approximately 4.4% of patients in the extension cohort over 3 years, with treatment-emergent adverse events decreasing from 92.1% in year 1 to 76.8% in year 3.

For ABBV-951, Aldred et al^[15]^ reported infusion-site events in >50% of patients, with cellulitis in 23% and abscess in 11.1%. Hallucinations occurred in 17.2% of patients. Jander et al^[16]^ reported ISRs in 25% of patients (6/24), with abscess in 8.3%. Notably, after implementing a 24-hour needle change protocol, skin reactions dropped from 50% in the first 10 patients to 7.1% in subsequent patients. Hartig et al^[17]^ reported any AE in 42.1%, local side effects in 22.8%, and visual hallucinations in 17.5%. Among those with local side effects (n = 13), 7 had mild erythema, 4 had moderate swelling/pain/phlegmon, and 2 had severe abscess or systemic inflammation.

Regarding the distinction between mild-to-moderate and severe ISRs – a critical point for clinical decision-making – mild-to-moderate reactions (erythema, pain, edema, pruritus, nodules without infection) comprised approximately 70% to 85% of all infusion-site events across studies. Severe reactions leading to treatment discontinuation occurred in 5% to 12% of patients (Fig. 1). Proactive management strategies, including 24-hour needle change protocols^[16]^ and structured patient education, significantly reduced severe reaction rates. Healthcare providers should prioritize training patients on infusion site rotation, aseptic technique, and early recognition of cellulitis to optimize treatment persistence.

Pinna et al^[18]^ reported mild-to-moderate cutaneous reactions in 22% of CSFLI patients, generally managed with simple measures or topical antibiotics, leading to therapy discontinuation in only 2 cases. Desjardins et al^[20]^ reported de novo apathy in 2 of 14 patients (14.3%). Cocco et al^[21]^ reported AEs in 87% of patients, primarily skin reactions, with cellulitis in 5 patients requiring oral antibiotics in 2. Hallucinations (new or worsened) occurred in 7 patients (30%), which is higher than previously reported and warrants clinical awareness in patients with cognitive vulnerability. Jost et al^[22]^ reported AEs in 55.2% of patients, with serious adverse events in 12.4%, hallucinations in 5.7%, and infusion site events in 5.7%.

### 3.7. Treatment discontinuation and retention

Treatment discontinuation rates varied across studies. For ND0612, the 3-year completion rate was 67.5%.^[14]^ For ABBV-951, Aldred et al^[15]^ reported 56.1% completion at 52 weeks, with 26.2% discontinuing due to AEs. Hartig et al^[17]^ reported that 33.3% of patients discontinued within 4 weeks, with 63.2% due to patient preference rather than AEs, highlighting the importance of patient education and expectation management. Pinna et al^[18]^ reported 10.7% discontinuation during the titration phase. Das et al^[19]^ reported a 26.5% discontinuation rate. Desjardins et al^[20]^ reported 0% discontinuation in patients switching from CSAI. Cocco et al^[21]^ reported 8.7% discontinuation (2/23). These findings suggest that patient selection, expectation management, and structured support are critical for treatment persistence.

Table 2 summarizes the outcomes and results from all included studies.

**Table 2:** Outcomes and results from included observational studies.

Study	Intervention	Follow-up	OFF time reduction	MDS-UPDRS III	MDS-UPDRS IV	PDQ-39/PDQ-8	Key adverse events	Discontinuation rate	Key notes
Ellenbogen et al 2025^[14]^	ND0612	36 mo	−2.81 h	NR	NR	NR	ISR 92.1% (year 1)	12%	67.5% completion; Good ON + 2.79 h
Aldred et al 2023^[15]^	ABBV-951	52 wk	−3.5 h	NR	NR	−7.9 to −10.6	ISR > 50%; hallucinations 17.2%; cellulitis 23%	26.2%	Morning akinesia 77.7%→27.8%; Good ON + 3.8 h
Jander et al 2025^[16]^	ABBV-951	9 wk	NR (OFF component −75%)	30→33 (NS)	−53% (*P* = .0267)	−16.6% (*P* = .0507)	ISR 25%; abscess 8.3%	16.7%	Skin reactions 50%→7.1% with 24-h needle change
Nordera 2025*	ABBV-951	3 mo	NR	Global improvement	Global improvement	NR	Mild ISRs; 1 severe skin infection	10%	Conference abstract; morning akinesia improved
Hartig et al 2026^[17]^	ABBV-951	Real-world	NR	−6.07 pts (*P* = .0008)	NR	NR	Any AE 42.1%; hallucinations 17.5%	10.5%	33.3% discontinued within 4 wk (63% preference)
Pinna et al 2025^[18]^	ABBV-951	3 mo	Both improved†	Both improved†	NR	Both improved† (PDQ-8)	Mild-moderate cutaneous 22%	10.7%	Greater LEDD increase vs LCIG; more follow-up visits
Das et al 2026^[19]^	ABBV-951	1 mo	−2.77 h	−11.37 pts	NR	−10.78 pts (PDQ-39)	ISR/infections	26.5%	First Canadian data
Desjardins et al 2025^[20]^	ABBV-951	3 mo	NR	54.5→NR (NS)	Significantly reduced (*P* = .011)	Improved (*P* = .0244) (PDQ-8)	De novo apathy 14.3%	0%	All switched from CSAI; 12/14 monotherapy
Cocco et al 2025^[21]^	ABBV-951	20 wk	−40% (*P* < .05)	39.1→35.2 (NS)	−34% (*P* < .05)	NR	Any AE 87%; hallucinations 30%	8.7%	Higher BMI = longer titration (*P* = .025)
Jost et al 2026^[22]^	ABBV-951	6 mo	−2.8 h (*P* ≤ .001)	−5.0 pts (*P* = .002)	NR	−5.6 pts (PDQ-39, *P* = .002)	AEs 55.2%; hallucinations 5.7%	NR	First multicountry real-world

AE = adverse event, BMI = body mass index, CSAI = continuous subcutaneous apomorphine infusion, CSFLI = continuous subcutaneous foslevodopa/foscarbidopa infusion, ISR = infusion-site reaction, LCIG = levodopa-carbidopa intestinal gel, LEDD = levodopa equivalent daily dose, NR = not reported, NS = not significant, PDQ-8 = Parkinson Disease Questionnaire-8, PDQ-39 = Parkinson Disease Questionnaire-39, UPDRS = Unified Parkinson Disease Rating Scale.

* Conference abstract.

† CSFLI and LCIG were similarly effective.

## 4. Discussion

This systematic review of 10 observational studies (total n = 867 patients) provides comprehensive real-world evidence that continuous subcutaneous levodopa infusion therapies – ND0612 and foslevodopa/foscarbidopa (ABBV-951) – are effective interventions for reducing motor fluctuations in APD. The magnitude of benefit is clinically meaningful, with sustained OFF time reductions of approximately 2.0 to 3.5 hours daily and corresponding increases in good-quality ON time, maintained for up to 36 months with ND0612 and 52 weeks with ABBV-951. These findings are remarkably consistent with recent RCT meta-analyses,^[10,11]^ confirming that efficacy observed in controlled trials translates effectively to real-world clinical practice.

### 4.1. ISRs: severity distinction and clinical implications

ISRs represent the primary tolerability challenge with subcutaneous levodopa administration, with overall incidence of 42% to 92% across observational studies. However, a critical finding from this review is that mild-to-moderate reactions comprise the majority (approximately 70%–85%) of events, while severe reactions leading to treatment discontinuation affect a minority (5%–12%) of patients. This distinction has important clinical implications. Mild-to-moderate erythema, pain, edema, and nodules can typically be managed conservatively with site rotation, topical preparations, and patient education, allowing continuation of effective therapy. Severe reactions – cellulitis requiring antibiotics (2%–6%) or refractory pain/nodules prompting discontinuation – represent the true barrier to long-term CSCI use. Importantly, Jander et al^[16]^ demonstrated that changing infusion needles every 24 hours (instead of every 72 hours) reduced skin reactions from 50% to 7.1%, suggesting that proactive management strategies significantly improve tolerability. These findings indicate that with appropriate patient selection, training, and ongoing support, most patients can successfully manage ISRs and derive sustained benefit from CSCI therapy.

### 4.2. Positioning of CSCI among device-aided therapies

CSCI therapy should be considered within the broader landscape of device-aided therapies for APD, which includes DBS, CSAI, and LCIG. Each modality offers distinct trade-offs:

CSCI (ND0612/ABBV-951) offers the advantage of being the least invasive device-aided therapy, requiring only subcutaneous catheter placement without surgical intervention. It can be initiated and discontinued readily, making it attractive for patients who are not candidates for DBS or who prefer a reversible option. Efficacy in reducing OFF time (approximately 2–3.5 hours) is comparable to LCIG and CSAI. However, ISRs are common (42%–92%), requiring ongoing skin management. The main trade-off is between low invasiveness and higher rates of local skin reactions.

DBS provides robust motor benefits and can be bilateral, with the advantage of being free from device-related skin reactions and allowing reduction of medication burden. However, DBS requires invasive neurosurgery with risks of intracranial hemorrhage (1%–2%), infection (5%–10%), and hardware complications. It is also contraindicated in patients with significant cognitive impairment or untreated psychiatric comorbidities. The trade-off is between superior efficacy for appropriate candidates and surgical risks.

CSAI (apomorphine) is an alternative subcutaneous infusion that has been available longer, with efficacy similar to CSCI for OFF time reduction. However, apomorphine carries risks of neuropsychiatric adverse effects (hallucinations, somnolence), nausea requiring concomitant domperidone, and hemolytic anemia requiring monitoring. The availability of CSCI with levodopa – the same molecule patients are already taking orally – may offer a more intuitive and acceptable option for many patients and clinicians. The trade-off is between established long-term safety data for apomorphine and the intuitive appeal of levodopa-based therapy.

LCIG requires surgical PEG-J tube placement and carries risks of procedural complications (peritonitis, tube dislodgement, stoma infection). While effective, LCIG is more invasive and less reversible than CSCI. Notably, Pinna et al^[18]^ demonstrated that while CSFLI and LCIG were similarly effective, CSFLI required more frequent follow-up visits and greater LEDD increases during titration, suggesting that LCIG may achieve optimization more efficiently but at the cost of greater invasiveness. CSCI offers a logical step between oral therapy and intestinal infusion, allowing patients to experience the benefits of continuous levodopa delivery without committing to surgical intervention.

### 4.3. Patient-provider discrepancy in perceived effectiveness

An important finding from Hartig et al^[17]^ was the discrepancy between clinician-rated (89%) and patient-rated (74%) effectiveness. A substantial proportion of patients (33.3%) discontinued treatment within 4 weeks, with 63.2% of discontinuations due to patient preference rather than AEs or ineffectiveness. Frequently communicated reasons included the size of the pump system, impairment of quality of life due to the device, and dissatisfaction with the magnitude of treatment effect. This finding underscores the critical importance of pretreatment counseling, realistic expectation setting, and shared decision-making. Clinicians should discuss lifestyle implications, device management requirements, and potential benefits versus burdens before initiating CSCI therapy.

### 4.4. Concordance between observational and RCT evidence

The concordance between observational and RCT findings strengthens confidence in the overall evidence base for CSCI therapies. While RCTs provide higher certainty evidence for causal inference with low risk of bias, observational studies in this review offer complementary insights: longer follow-up (up to 36 months vs 4–12 weeks in most RCTs), larger and more diverse patient populations in some studies, real-world effectiveness data including patients with comorbidities typically excluded from RCTs, and practical information on ISR management and long-term adherence. The similar magnitude of OFF time reduction between RCTs (1.98 hours from Burton et al^[10]^) and observational studies (2.0–3.5 hours) provides strong convergent validity for the efficacy of CSCI therapies.

### 4.5. Limitations and global applicability

Several limitations of this review must be acknowledged. First, observational studies are subject to selection bias, lack of blinding, and potential confounding. Second, the included studies are predominantly from North America, Europe, and Israel, with no data from low- and middle-income countries (LMICs). This is a critical limitation given that the global burden of PD (6.1 million individuals) is substantial in LMICs, where healthcare access, device availability, and patient comorbidity profiles may differ significantly. Subcutaneous infusion device costs, pump availability, access to specialized nursing support, and reliable medication supply chains may be prohibitive in many LMIC settings. Third, heterogeneity in outcome measurement and reporting across studies limited our ability to perform meta-analysis. Fourth, long-term safety data beyond 36 months remain limited. Fifth, while we quantified severe versus mild-to-moderate ISRs, not all studies provided granular severity data. Sixth, publication bias cannot be excluded, though the consistent direction of effects across studies makes substantial bias less likely.

### 4.6. Implications for clinical practice

For appropriately selected patients with APD and persistent motor fluctuations despite optimized oral therapy, continuous subcutaneous levodopa infusion offers an efficacious and generally well-tolerated therapeutic option. Ideal candidates include those with documented motor fluctuations (≥2–3 hours daily OFF time significantly impacting quality of life), good cognitive function, absence of active psychosis, adequate manual dexterity or caregiver support for device management, sufficient subcutaneous tissue for infusion, and realistic expectations regarding ISRs.

The choice between available subcutaneous formulations should be individualized. ABBV-951 may offer advantages in terms of lower rates of ISRs, particularly erythema and nodules, potentially translating into better long-term tolerability. Its Food and Drug Administration-approved status (October 2024) also facilitates access. However, both formulations appear similarly efficacious, and ND0612 remains a viable option where available.

Multidisciplinary care is essential for optimizing outcomes. Dedicated training for patients and caregivers on infusion site management, routine site rotation (every 24–48 hours), early recognition of cellulitis, and access to wound care specialists can significantly reduce severe reaction rates and improve treatment persistence.

### 4.7. Future research directions

Future research priorities should include: head-to-head comparative trials between subcutaneous formulations to definitively differentiate ND0612 and ABBV-951; extended safety surveillance beyond 36 months, particularly for infusion-site sequelae with prolonged use; studies in LMIC settings to assess feasibility, cost-effectiveness, and cultural adaptations; identification of predictors of response (clinical, genetic, or biomarker-based) to enable personalized treatment selection; comparative effectiveness research directly comparing CSCI with DBS, CSAI, and LCIG within the same study populations; development of standardized severity grading for ISRs to facilitate cross-study comparisons; and investigation of strategies to improve patient acceptance and reduce preference-driven discontinuation.

## 5. Conclusions

Continuous subcutaneous levodopa infusion therapies represent an important advance in the management of APD with motor fluctuations. Real-world observational studies demonstrate sustained efficacy for up to 36 months, with OFF time reductions of approximately 2.0 to 3.5 hours daily and corresponding improvements in quality of life. ISRs are common (42%–92%) but are predominantly mild-to-moderate (70%–85% of events), with severe reactions leading to discontinuation affecting a minority (5%–12%) of patients in routine clinical practice. Proactive management strategies, including 24-hour needle change protocols, significantly reduce severe reaction rates. These findings complement RCT evidence and support CSCI as an effective, less-invasive alternative to intestinal levodopa infusion. For appropriately selected patients, CSCI offers a valuable treatment option that can significantly improve motor fluctuations and quality of life while avoiding surgical intervention. However, the absence of data from LMICs represents a critical gap that must be addressed in future research to ensure global applicability of these findings.

## Author contributions

**Conceptualization:** Waqar Ahmed Cheema, Muhammad Asad Shabbir.

**Data curation:** Muhammad Shamoon, Muhammad Talha.

**Formal analysis:** Dansih Yousuf.

**Investigation:** Anousha Tanveer, Labeeba Abdul Ghafoor.

**Project administration:** Fiza Nisar.

**Methodology:** Wajeeha Asif, Labeeba Abdul Ghafoor.

**Software:** Mukesh Sharma.

**Writing – original draft:** Aasim Ali, Waqar Ahmed Cheema, Hammad Azam.

**Writing – review & editing:** Muhammad Abdullah Ali, Usama Saleem, Armaghana Abdullah.
